# Anti-Inflammatory and Anti-Aging Evaluation of Pigment–Protein Complex Extracted from *Chlorella Pyrenoidosa*

**DOI:** 10.3390/md17100586

**Published:** 2019-10-16

**Authors:** Ruilin Zhang, Jian Chen, Xinwu Mao, Ping Qi, Xuewu Zhang

**Affiliations:** 1 College of Food Science and Engineering, South China University of Technology, Guangzhou 510640, China; ruilin_zhang92@163.com (R.Z.); fejchen@scut.edu.cn (J.C.); 2 Era (China) Company Ltd., Shenzhen 518115; China; 3 Guangzhou Institute for Food Inspection, Guangzhou 511400, China; gzsp2000@163.com

**Keywords:** *Chlorella pyrenoidosa*, pigment–protein complex, anti-inflammation, anti-aging, NF-κB, PPARs

## Abstract

Oxidative stress contributes to chronic inflammatory processes implicated in aging, referred to as “inflamm-aging.” In this study, the potential anti-inflammatory and anti-aging effects of a pigment–protein complex (PPC) from *Chlorella pyrenoidosa* were investigated using lipopolysaccharide (LPS)-stimulated RAW 264.7 macrophages and D-galactose (D-gal)-induced aging in a murine model. Results indicated that PPC inhibits the production of the inflammatory cytokines TNF-α and IL-6, and the inflammatory mediator nitric oxide (NO) in LPS-stimulated RAW 264.7 cells. It also protected mice from D-gal induced informatory aging by increasing the activity of the antioxidant enzyme, such as superoxide dismutase (SOD), inhibiting D-gal-induced NF-κB upregulation, and increasing PPARs expression in the brain and gut. The findings indicated that PPC has favorable anti-inflammatory and anti-aging properties, and could be useful in the treatment of acute inflammation and senescence diseases.

## 1. Introduction

Cellular senescence, a signal of aging, is stimulated by many different stress conditions, including oxidative, radiative, and inflammatory stress. Both oxidative stress and inflammation contribute to monolithic aging [1]. Senescent cells secrete inflammatory cytokines, growth factors, and reactive oxygen species (ROS); these molecules contribute to cellular inflammation and oxidative stress, which in turn increase inflammatory cytokines and ROS, resulting in a deleterious cascade [2]. “Inflamm-aging” is a complex, systemic biological response to harmful stimuli and injury [3] characterized by the combined activities of leukocytes and macrophages, which help the body resist inflammatory stimuli and repair damage. Lipopolysaccharide (LPS) is a bacterial compound that activates macrophages and stimulates their release of pro-inflammatory cytokines, such as interleukin (IL)-1β, IL-6, and tumor necrosis factor (TNF)-α, as well as inflammatory mediators, such as nitric oxide (NO), during oxidative stress. D-galactose (D-gal), a nutrient usually obtained from lactose in milk, has been used in previous studies to induce aging in mice through abnormal metabolism [4,5]. Both LPS and D-gal can cause oxidative stress and mitochondrial dysfunction, and damaged mitochondria generate reactive oxygen species (ROS) that have been implicated in inflamm-aging; in addition, increased ROS could lead to additional mitochondrial damage and accelerate the inflamm-aging process. 

Evidence indicates that inflammatory signaling stimulates O2 production and oxidative stress via nuclear factor κB (NF-κB), p53, and p21, all of which can induce or facilitate age-related inflammation [1,2,6]. NF-κB activates the transcription of pro-inflammatory cytokines, and oxidative stress and DNA damage have been shown to upregulate the p53/p21 pathway and trigger the secretion of senescence-associated secretory phenotype (SASP) components by increasing NF-κB activity [7]. Other inflammation-sensitive pathways have also been implicated in aging. While existing anti-inflammatory medications such as glucocorticoids and nonsteroidals (NSAIDs) can significantly suppress the production of pro-inflammatory cytokines [2,8], the side effects of these drugs include immune system depression and gastrointestinal ulcerations [8]. The development of other agents to slow the inflamm-aging process, particularly those from natural sources with fewer adverse effects, remains necessary.

Marine microalgae are a promising sustainable source of bioactive compounds, and several studies have reported on the anti-inflammatory activity of peptides, proteins, and polysaccharides in these microorganisms [9,10,11]. A pigment–protein complex (PPC) thereof is the most abundant source of light-harvesting protein complexes located in algal thylakoid membranes that manage photosynthesis and chlorophyll photoprotection [12]. Although many experiments have isolated and analyzed the PPC composition and structure from marine microalgae such as *Chlorella vulgaris* and *Codium fragile* [12,13], little is known about the potential bioactivities and pharmacological mechanisms of PPC extracts. In this study, we derived PPC from *Chlorella pyrenoidosa* through aqueous and spirituous soaking and gel filtration to investigate the potential anti-aging and anti-inflammatory effects of this extract, and explore their underlying mechanisms. The inflamm-aging-related regulation of IL-β, IL-6, TNF-α, and inducible NO synthase, as well as the impacts on the expression of NF-κB, PPARα, PPARγ, p21, and p53, were thereby evaluated.

## 2. Results

### 2.1. Isolation and Characterization of PPC

**Figure 1:**
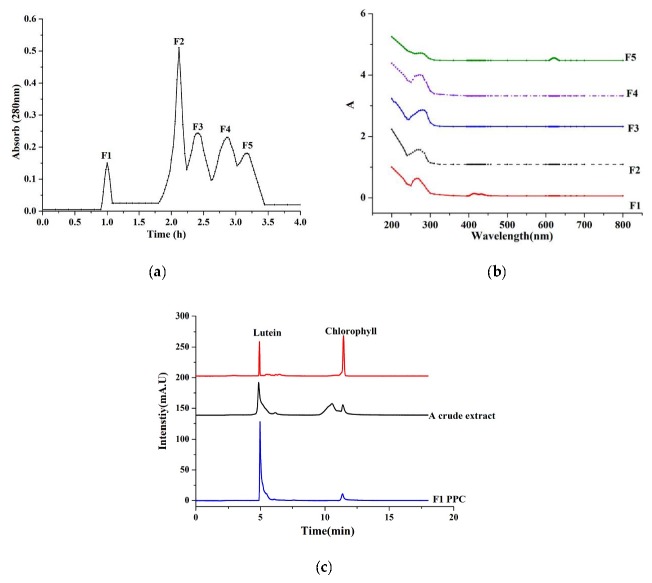
Purification and characterization of the pigment–protein mixture from *Chlorella pyrenoidosa*. (**a**) Sephadex G-25 gel filtration chromatography; (**b**) Absorption spectrum of the pigment–protein complex (PPC); (**c**) HPLC analysis.

A crude pigment–protein extract from *Chlorella pyrenoidosa* was obtained by aqueous and spirituous soaking extraction [14]. Previous studies have reported the presence of carotenoid and chlorophyll pigments in other crude pigment–protein extracts [14,15]. The crude extract of pigment-protein was separated by a Sephadex G-25 column, and fractions F1 through F5 all contained protein (Figure 1a). Peaks were observed at absorbances of 400 to 450 nm and 610 nm (Figure 1b), indicating significant amounts of pigment in F1 and F5 fractions. However, the bicinchoninic acid (BCA) protein data revealed that F5 only contained a small amount of protein (Table 1). HPLC analysis of the crude pigment–protein extract and the F1 purified pigment–protein complex confirmed that lutein and chlorophyll were the predominant pigments in the crude extract (Figure 1), with lutein predominant in F1. 

### 2.2. PPC Effects on RAW 264.7 Cell Viability

**Figure 2:**
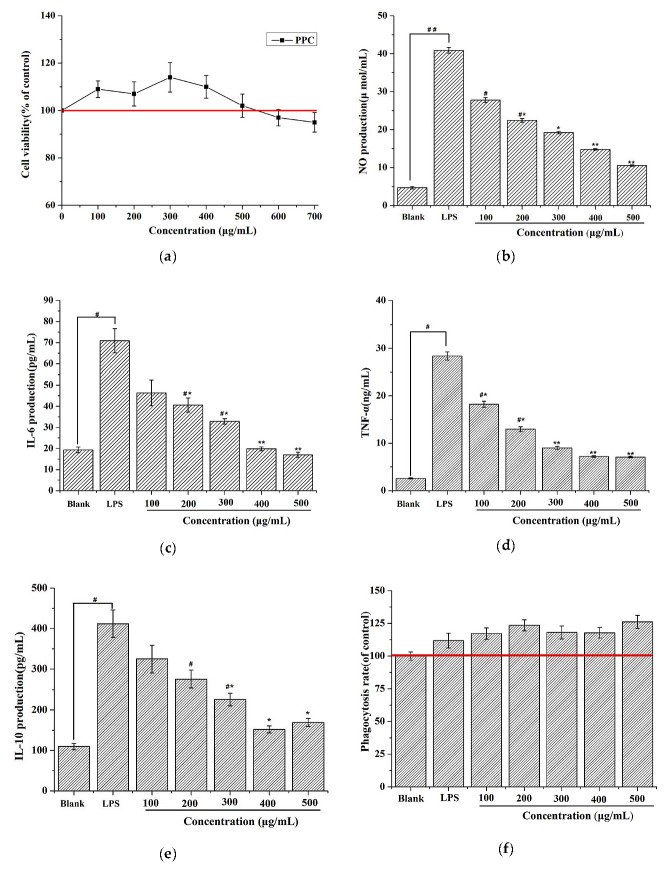
Effect of PPC on RAW 264.7 cells. (**a**) The viability was measured by 3-(4,5-dimethylthiazol-2-yl)-2,5-diphenyltetrazolium bromide (MTT) assay. The control group consisted of untreated cells and was considered as 100% of viable cells. Results were expressed as a percentage of viable cells when compared with the control group. (**b**) Nitric oxide (NO) production levels; (**c**) Expression levels of tumor necrosis factor (TNF)-α; (**d**) The levels of interleukin (IL)-6 production; (**e**) The levels of IL-10 production; (**f**) Phagocytosis rate. All the experiments were performed in triplicate. Duncan’s new multiple range test was performed to determine the significance differences. The values are expressed as the as mean ± SD. ^#^ *p* < 0.05 vs. the blank group, ^##^ *p* < 0.01 vs. the blank group, * *p* < 0.05 vs. the LPS group, and ** *p* < 0.05 vs. the lipopolysaccharide (LPS) group.

Figure 2a shows a 100% cell survival rate for PPC at concentrations ranging from 0 to 500 μg/mL, indicating that PPC was not toxic to RAW 264.7 cells at these concentrations. Thus, these concentrations were used in subsequent assays.

### 2.3. In Vitro Anti-Inflammatory Activity

The anti-inflammatory effects of PPC were tested by stimulating RAW 264.7 cells with 2 μg/mL of LPS, and then treating them with the indicated concentrations of PPC. TNF-α levels in the model (LPS-stimulated cells) supernatant increased from 2.6 ± 0.13 ng/mL to 28.09 ± 0.64 ng/mL (Figure 2). The addition of PPC decreased levels of TNF-α at all concentrations in a dose-dependent manner, with the most pronounced reduction, to 7.56 ± 0.19 pg/mL, observed at a concentration of 500 μg/mL. LPS stimulation resulted in an increased production of IL-6 (70.9 ± 5.94 versus 19.36 ± 1.34 pg/mL) and NO (40.9 ± 1.74 versus 4.66 ± 0.14 μmol/mL) in macrophages over the control group. After supplementation with PPC, amounts of IL-6 and NO decreased at all concentrations, and the most pronounced reductions of IL-6 to 20.5 ± 2.81 pg/mL and NO to 10.55 ± 0.21 μmol/mL were observed at 500 μg/mL, indicating a dose-dependent reduction of inflammatory cytokines in LPS-stimulated macrophages cells, with the most pronounced effect observed at 500 μg/mL. Previous research showed that a reduction in anti-inflammatory cytokines and IL-10 contribute to the immunomodulation [16,17]. The most significant reductions to IL-10 secretion and phagocytosis were observed at 400 μg/mL PPC. The data showed that PPC administration reduces in NO, IL-6, and IL-10 release in LPS-stimulated murine macrophages, indicating that PPC has immunomodulation and anti-inflammatory effects.

### 2.4. PPC Effects on KMB-17 Cell Viability

**Figure 3:**
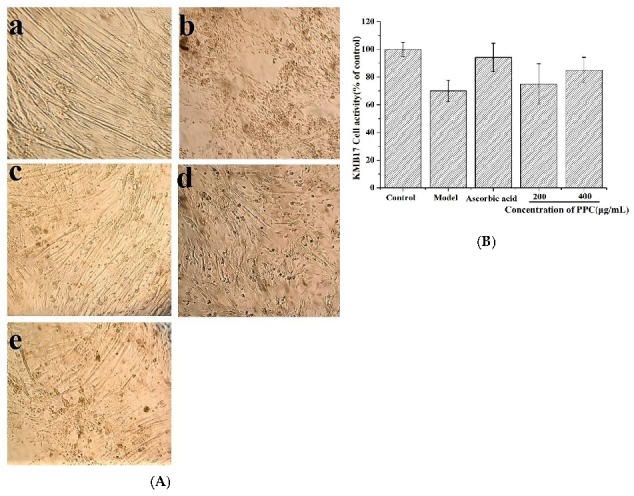
D-galactose (D-gal) induced KMB17 cells premature senescence in vitro. (**A**) Cells morphology changes: normal group (**a**), Model group D-gal-stimulated (**b**), Ascorbic acid group (**c**), 200 μg/mL PPC (**d**), 400 μg/mL PPC (**e**); (**B**) The effects of PPC on KMB17 cell viability. The experiments were performed in triplicate, and the values are expressed as the as mean ± SD. Results were expressed as a percentage of viable cells when compared with the control group.

The human embryonic diploid lung fibroblast cell KMB17 is one of the classical experimental models for studying cellular senescence. Cellular aging is characterized by altered cellular morphology such as decreased cell proliferation and changes in cell size during the culturing process [16]. Currently, it is reported that many agents (hydrogen peroxide, radiation, and D-gal) can be good inducers, causing oxidative stress and leading to cellular senescence [18]. In the study, KMB-17 cells were stimulated with 2 μg/mL of D-gal, and then treated with stepped concentrations of PPC or 20 μg/mL of ascorbic acid. The morphology of the model cells was poorly stimulated with 2 μg/mL of D-gal (Figure 3A), indicating that the state of senescence has been established. Intriguingly, PPC treatment ameliorated this loss of morphology significantly at 400 μg/mL, although to a lesser degree than ascorbic acid. Meanwhile, cell viability increased by PPC administration, which confirmed PPC’s viability to alleviate aging (Figure 3B). 

### 2.5. PPC Effects on Antioxidation and Peroxidation in Guts of D-Gal-Treated Mice

To investigate whether the PPC-associated anti-aging effects in mice were related to altered antioxidant and anti-inflammatory capacities, levels of important antioxidant enzymes and inflammatory cytokines in mouse gut tissue were assessed. Levels of the antioxidant enzyme superoxide dismutase (SOD), which enhances antioxidant defenses and anti-inflammatory processes, decreased significantly in D-gal-treated mice compared to controls. Conversely, levels of malondialdehyde (MDA), an indicator of oxidative stress, were significantly higher in gut tissues from D-gal-treated mice than those from control mice (*p* < 0.05). Higher concentrations of PPC were associated with increases in SOD activity and attenuation of the MDA upsurge caused by D-gal induction (*p* < 0.05). However, lower concentrations of PPC were not associated with a significant decrease in SOD in D-gal-treated mice.

**Figure 4:**
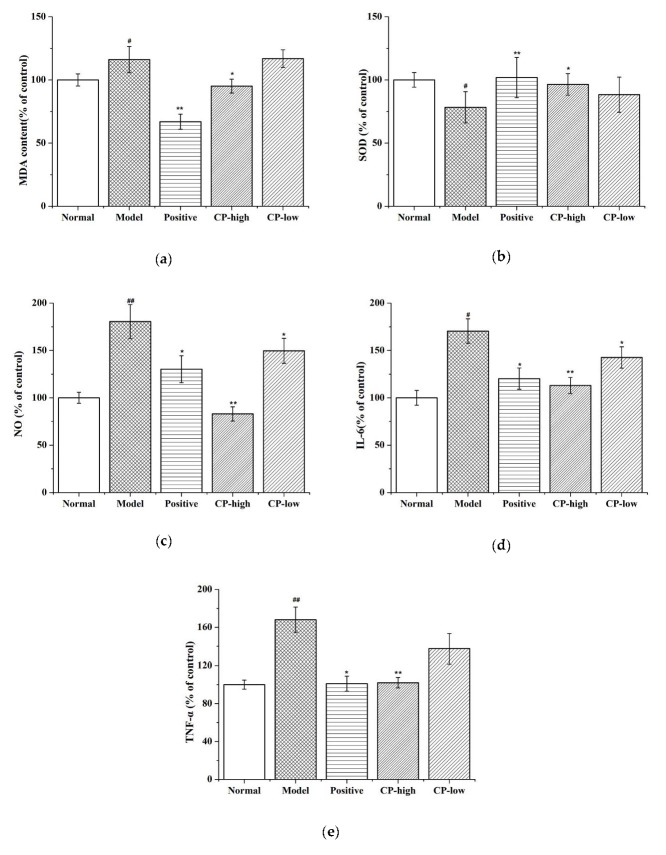

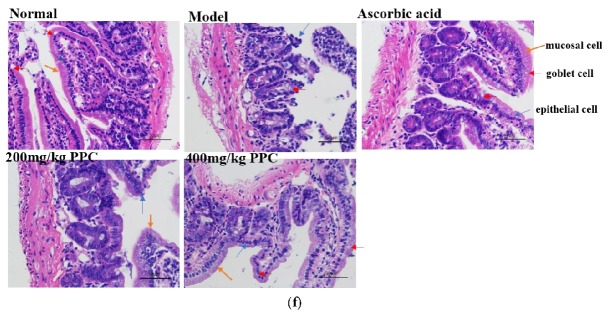
Effect of PPC on superoxide dismutase (SOD), malondialdehyde (MDA), NO, IL-6, TNF-α levels, and the morphological features of gut in D-gal-induced mice. (**a**) Changes in MDA, (**b**) SOD, and (**c**) NO production levels; (**d**) The levels of IL-6 production; (**e**) Expression levels of TNF-α; (**f**) The morphological features of hematoxylin and eosin (H&E) stained gut sections, Scale bar = 50 μm. A one-way ANOVA was performed to compare the three experimental groups. All the data are mean ± SD of three independent experiments. ^#^ *p* < 0.05 vs. the control group, ^##^ *p* < 0.01 vs. the control group, * *p* < 0.05 vs. the model group, and ** *p* < 0.01 vs. the model group.

Figure 4 presents the activity of inflammation-related indices, including inflammatory cytokines and the pro-inflammatory mediator NO, in experimental mice. D-gal-stimulated (model group) mice produced more NO, TNF-α, and IL-6 than negative (non-D-gal) controls. However, higher concentrations of PPC were associated with decreased levels of NO, TNF-α, and IL-6 in D-gal mice. Ascorbic acid also reduced NO, TNF-α, and IL-6 levels compared to the model group; however, to a lesser degree than high concentrations of PPC.

### 2.6. Histological Results of PPC Treatment in Guts of D-gal-Treated Mice

Morphological features of stained hematoxylin and eosin (H&E) gut tissue sections from model mice showed significant damage to gut cell structures compared to control mice (Figure 4f). In D-gal-treated mice, gut damage was extensive, as evidenced by the presence of necrotic epithelial and intestinal mucosal cells. In certain areas, intestinal mucosal cells were observed in the tubular lumen, accompanied by near-total goblet cell loss. In the group treated with higher concentrations of PPC, goblet cell loss was attenuate, and epithelial cells appeared largely undamaged, indicating a decrease in inflammation in this group. However, mice treated with low concentrations of PPC, still displayed marked epithelial crypt cell hyperplasia and goblet cell loss across epithelial cell layers. Figure 4e shows there are better complete epithelial cell structures and abundant goblet cells in mice treated with the highest concentrations of PPC. 

### 2.7. PPC Effects on Expression of IL-10, NF-κB, PPARα, PPARγ, p53, and P21

**Figure 5:**
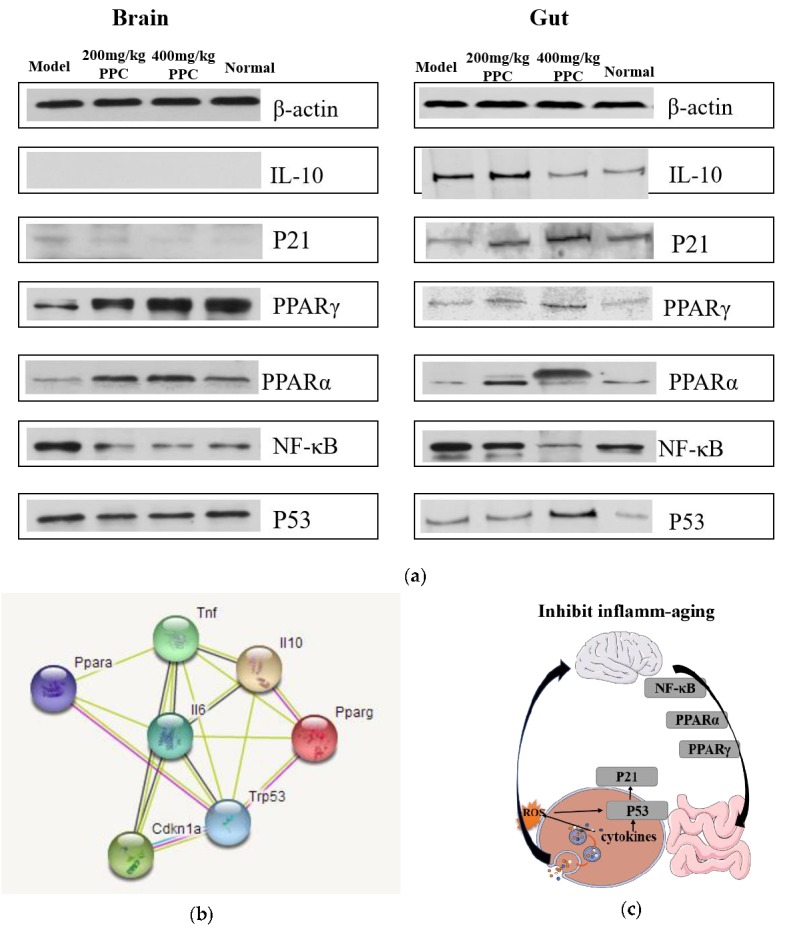
(**a**) Effects of PPC on the expression levels of nuclear factor κB (NF-κB), PPARα, PPARγ, and p53 in mice, the proteins of brain and gut in each group were processed by Western blotting, and all data performed in triplicate; (**b**) Potential regulation pathways by Search Tool for the Retrieval of Interacting Genes/Proteins (STRING) software (http://string.embl.de/); (**c**) Proposed mechanism of action on brain and intestine.

The mechanisms underlying the anti-aging properties of PPC in D-gal-treated mice were assessed by Western blot analysis of mice brain tissue levels of NF-κB, PPARα, PPARγ, p53, and p21. Mice receiving 400 mg/kg per day of PPC demonstrated a significant increase in PPARα and PPARγ expression compared to the D-gal model and control groups (Figure 5), underscoring results observed in the gut. PPC markedly inhibited the expression of NF-κB in the gut and brains of D-gal-treated mice. However, results for p21 and p53 in the brain and gut were inconclusive. In D-gal + PPC mice, the level of p21 in the brain was slightly increased over the model group, which was a difference that was not significant and was in opposition to the marked signal intensity thereof over time in mice gut samples from this group. Expression levels of p53 varied little across groups, while the signal was the strongest in the gut samples from the high PPC group. Western blot analysis also showed that NF-κB expression increased in the model group, and that PPC treatment cut this increase in half. Levels of IL-10 in the brain and gut indicated that D-gal was not enough to cause inflammatory injury in the brain, although levels were increased in the gut, and PPC rescued this increase. The functional and predicted associations for the differentially expressed cytokines and proteins involved in the inflammatory-aging process in the animal model were induced by D-gal (Figure 5b). 

## 3. Discussion

In the process of senescence, the functionality of cells and tissues becomes markedly impaired, and oxidative stress and a weakened immune system are characteristic signs of senescence. In the different aging models, increased amounts of other cellular senescence markers such as ROS level, disordered immune system, and poor cell morphology have been observed [18,19]. Previous studies have implicated reactive oxygen species (ROS) in inflammatory and aging processes [18,19]. In this study, the anti-aging effects of PPC were evaluated in a reliable aging model induced by D-gal. The biochemical and physiological changed in the D-gal-induced aging processes include increases in senescence, oxidative stress, mitochondrial dysfunction, and apoptosis; therefore, the model very much represents the natural aging cell and animal models [18]. Our results suggested that PPC increased cell proliferation and ameliorated cell morphology damage caused by D-gal in KMB17; PPC could improve immune cells function by increasing their anti-inflammatory capacity and increasing the antioxidant enzymes activities to ameliorate the inflammation associated with the aging process. 

PPC administration diminished oxidative damage, decreased MAD activity, and increased SOD activity in a rat model. It has been shown that D-gal increases peroxidation and SOD activity while decreasing catalase and glutathione peroxidase directly or via activation of the NF-κB pathway [20]. In an animal study, the inflammation- and senescence-associated proteins NF-κB, PPARα, PPARγ, p53, and p21 were measured by Western blot to elucidate potential mechanisms of PPC. Previous studies have indicated that the master regulator nuclear factor NF-κB is crucial in inflammatory responses and cellular senescence [21], and many effective drugs could alleviate inflammation-aging by upregulated NF-κB expression. Our results show that compared with controls, the levels of NF-κB were prominently increased in the model mice, but effectively reverted in mice treated with PPC; therefore, it can be interpreted that PPC could potentially regulate and maintain the cellular homeostasis with respect to D-gal induced inflammation and oxidative stress. PPARs are another family of regulators implicated in senescence-associated pathophysiological processes related to energy metabolism and oxidative stress [20,21]. The evidence supports an interrelation among ROS, PPARs, and NF-κB. According to early research, ROS facilitate the activation of the NF-κB pathway by inhibiting the phosphorylation of IκBα [22]. Then, activated NF-κB negatively regulates the PPARs response to an oxidative stress environment [23]. The data shows that the strongest NF-κB signal was accompanied with the weakest signals of PPARα and PPARγ in the model group; the opposite was in the high-dose PPC group, which is consistent with the research.

The p53/p21 pathway is not only the primary mediator of cellular senescence, but also essential for the cellular damage response pathways [19]. The pathway potentiates inflammatory responses and inhibits both apoptosis and proliferation, leading to cellular senescence [20]. The literature shows that the regulation of anti-aging occurs by activating the pathway p53/p21. However, in our results, levels of p21 and p53 in the brain and gut were somewhat inconclusive, with the strongest intensities of p21 and p53 in high concentration in the PPC gut, and little change in the PPC brain. We surmise that PPC may exert its protective effect against age-associated inflammatory factors by downregulating the p53/p21 pathway in the gut, if not in the brain. 

STRING-based network analysis revealed that the cytokines TNF-α, IL-6, and IL-10, and proteins PPARa, PPARg, p53, and p21 were mapped to a network (Figure 5b) that directly relates to inflammatory and aging processes, including inflammatory bowel disease (IBD) and cellular senescence. Cancer, viral infection, non-alcoholic fatty liver disease (NAFLD), and insulin resistance pathways were also involved, suggesting that PPC efficacy may extend to other conditions. Overall, the present data suggest that PPC can slow down the inflamm-aging process by lowering NF-κB and PPARs signaling in the brain and gut, as well as activating the p53/p21 pathway in the intestine (Figure 5c); however, further studies are needed to confirm this.

## 4. Materials and Methods

### 4.1. Materials and Chemicals

*C. pyrenoidosa* powder (62.4% ± 1% total protein content) was obtained from Dr. Zhang Daojing at the East China University of Technology, Shanghai, China. Bicinchoninic acid (BCA) protein, superoxide dismutase (SOD), and malondialdehyde (MDA) kits were obtained from the Nanjing Jiancheng Bioengineering Institute (China). LPS and 3-(4,5-dimethylthiazol-2-yl)-2,5-diphenyltetrazolium bromide (MTT) were purchased from Sigma-Aldrich (St. Louis, MO, USA). Primary and secondary antibodies were purchased from Cell Signaling Technology (Danvers, MA, USA). Methanol and acetonitrile used in liquid chromatography were of HPLC grade. All other chemicals and reagents were also of analytical grade and commercially available.

### 4.2. Pigment–Protein Complex (PPC)

Crude pigment–protein mixture was derived as described in previous studies [14], and the extracted pigment–protein complement was then lyophilized (FDU-1200, Tokyo Rikakikai Co., Ltd., Tokyo, Japan). The protein content therein was evaluated with a BCA kit (Nanjing Jiancheng Bioengineering Institute, China), and the extraction ratio was calculated.

### 4.3. Gel Filtration Chromatography

The fraction with the highest radical-scavenging activity was separated by gel filtration chromatography. Two milliliters of bioactive fraction dissolved in distilled water at a concentration of 20 mg/mL were loaded onto a Sephadex G-25 (1.6, 3 × 30 cm). The column was eluted with distilled water at a flow rate of 0.5 mL/min. The absorbance of the collected solution was evaluated at 280 nm. All eluates with the same peak were combined and freeze-dried. 

### 4.4. Characterization of Pigment–Protein Complex

#### 4.4.1. Spectrophotometric Measurement

Absorption spectra of the pigment–protein fractions were recorded over a wavelength range of 200 to 800 nm on a Shimadzu UV-2550 spectrophotometer at room temperature. 

#### 4.4.2. Pigment Determination

Five volumes of methanol were added to the protein solution, and the precipitate was subsequently removed. For high-performance liquid chromatography (HPLC) analysis, the PPC was eluted with a Shimadzu LC-20A system equipped with a Unimicro SP-120-5-C18 (4.6 × 180 mm, 3 μm) column. The mobile phase contained variational proportions of acetonitrile/ultrapure water (from 0:100% to 90%:10%), and absorbances were monitored at a wavelength of 450 nm. 

#### 4.4.3. Protein Determination

The protein content of the fractions was quantified with the BCA kit in accordance with the manufacturer’s instructions.

### 4.5. Cell Culture

RAW 264.7 macrophages were maintained at 37 °C in a humidified atmosphere of 5% CO_2_ in Dulbecco’s modified Eagle’s medium (DMEM; Invitrogen) supplemented with 10% fetal bovine serum and 1% penicillin and streptomycin solution. The KMB17 cell line (purchased from the Institute of Laboratory Animal Science, Jinan University), which originated from a human embryonic lung, is now widely used for cell senescence evaluations in vitro. KMB17 cells were grown in DMEM supplemented with 10% fetal bovine serum.

#### 4.5.1. PPC Toxicity Tests

RAW 264.7 cells were seeded in 96-well plates at a density of 2 × 10^5^ cells per well. After 24 h, the medium was exchanged for that with stepped concentrations of PPC, and cells were incubated for another 24 h. Then, 20 μL of MTT (1 mg/mL) was added to each well, and cells were incubated for another 4 h at 37 °C. The supernatant was subsequently decanted, and 180 μL of dimethyl sulfoxide (DMSO) was added to the cells. Then, absorbances were measured with a microplate reader at a wavelength of 570 nm.

#### 4.5.2. Measurement of Cytokines and NO Production

RAW 264.7 cells were plated into 96-well plates at 2 × 10^5^ cells per well and stimulated with 2 μg/mL LPS for 24 h. Then, cells were treated with stepped concentrations of PPC for 24 h, followed by collection of the culture medium. Levels of TNF-α, IL-6, and IL-10 in the supernatants were assessed with enzyme-linked immunosorbent assay (ELISA) kits according to the manufacturer’s instructions. 

#### 4.5.3. Phagocytosis rate

RAW 264.7 cells (1 × 10^5^ cells/well) were plated on 96-well plates and stimulated with 2 μg/mL LPS for 24 h. Then, cells were treated with stepped concentrations of PPC for 24 h followed by supplementation with 100 μL of 0.075% neutral red saline solution and subsequent incubation for 1 h at 37 °C. Then, cells were washed three times with PBS, and 100 μL of cell lysate was added into each well. After 2 h, absorption values were measured at 540 nm, and the phagocytosis rate was calculated with the following formula:

(1) $$\text{Phagocytosis rate}\%=\frac{ODa-OD0}{ODb-OD0}\times 100\%$$

where *ODb* refers to the value of the treated wells, *OD*0 is the value of a blank well, and *ODa* is the value of the control well.

#### 4.5.4. KMB-17 Cell Senescence in Vitro

KMB-17 cells were seeded in 6-well plates at a density of 5 × 10^3^ cells per well and stimulated with 2 μg/mL D-gal. After 24 h, the medium was exchanged for that with stepped concentrations of PPC followed by incubation for another 24 h. Then, cells were observed with light microscopy, and cell viability was measured.

### 4.6. Murine Model

A total of 30 C57BL/6 male and female mice (50%/50%) were randomly divided into five groups (*n* = 6). In the D-gal-administration group (model group), the mice were intraperitoneally (IP) injected with 120 mg/kg D-galactose once daily for 30 days. In the D-gal + PPC group, mice were IP injected with D-gal combined with either 200 or 400 mg/kg of PPC once daily for 30 days. In the control group, mice received IP saline at the same dose and time as their experimental counterparts. In the ascorbic acid group, mice were injected with D-galactose and 40 g/kg ascorbic acid once daily for 30 days. On the 31st day, the mice were anesthetized with sodium pentobarbital and euthanized by cervical dislocation. Then, brain and gut samples from the mice were collected for further analysis. The study was completed at the Institute of Laboratory Animal Science. The protocol was approved by the Laboratory Animal Ethics Committee Jian University on 2 April 2018 (the project identification code is 20180402-08).

### 4.7. Histological Analysis

Gut tissues were fixed in fresh 4% paraformaldehyde (pH 7.4) for 3 days and then embedded in paraffin. The sections were cut and stained with hematoxylin and eosin (H&E) and examined under light microscopy (Axioskop 40, Zeiss, Germany).

### 4.8. Measurement of Enzyme Levels

Levels of SOD and MDA were analyzed with the appropriate enzymatic kits, and protein content was measured with a BCA assay kit. Levels are expressed as nmol per mg of protein.

### 4.9. Western Blot Analysis

Brain and gut tissues were washed and lysed in RIPA (Radio Immunoprecipitation Assay) buffer for 30 min, and the lysates were centrifuged at 12,000× *g* for 25 min at 4 °C. Supernatants were decanted, mixed with 4X SDS sample buffer, and boiled for 10 min. Samples were run through a 4%/15% SDS polyacrylamide gel and then transferred to a polyvinylidene difluoride (PVDF) membrane. The membrane was blocked with 5% (*w/v*) BSA for 30 mins, and then incubated with primary antibody at 4 °C for 12 h. After 3 washings with TBST (Tris-Buffered Saline Tween-20), the membrane was incubated with secondary antibody for 1 h. The membrane was again washed with TBST, and bands were detected with a microplate scanner (Thermo Fisher Scientific, Inc.). Fluorescence intensities were measured using ImageJ software (National Institutes of Health, Bethesda, MD, USA).

### 4.10. Statistical Analysis

To ensure the reliability and credibility of the results, all experiments were performed in triplicate and independently repeated at least three times. Quantitative data are expressed as mean ± standard deviation. One-way ANOVA or Duncan’s new multiple range tests were conducted to test for significance using SPSS 10.0 software. A *p*-value of less than 0.05 was considered statistically significant.

## 5. Conclusions

In the study, the PPC-protected morphology of stem cell KMB17 against D-gal induced aging; in addition, it ameliorated the D-gal-induced through the reduction of intracellular ROS and inflammatory cytokines production. These suggest that PPC may be a candidate for the aging treatment. 

## Figures and Tables

**Table 1:** The protein content in the pigment–protein mixture fraction.

Groups	Protein Content (%)
F1	62.83 ± 3.78%
F5	1.92% ± 0.2%

The vales are expressed as averages ± standard deviation, *n* = 3.
